# Altered embryonic development in northern bobwhite quail (*Colinus virginianus*) induced by pre-incubation oscillatory thermal stresses mimicking global warming predictions

**DOI:** 10.1371/journal.pone.0184670

**Published:** 2017-09-19

**Authors:** Kelly S. Reyna, Warren W. Burggren

**Affiliations:** Developmental Integrative Biology Research Group, Department of Biological Sciences, University of North Texas, Denton, Texas, United States of America; Gaziosmanpasa Universitesi, TURKEY

## Abstract

Global warming is likely to alter reproductive success of ground-nesting birds that lay eggs normally left unattended for days or even weeks before actual parental incubation, especially in already warm climates. The native North American bobwhite quail (*Colinus virginianus*) is such a species, and pre-incubation quail eggs may experience temperatures ≥45°C. Yet, almost nothing is known about embryonic survival after such high pre-incubation temperatures. Freshly laid bobwhite quail eggs were exposed during a 12 day pre-incubation period to one of five thermal regimes: low oscillating temperatures (25–40°C, mean = 28.9°C), high oscillating temperatures (30–45°C, mean = 33.9°C), low constant temperatures (28.85°C), high constant temperatures (mean = 33.9°C), or commercially employed pre-incubation temperatures (20°C). After treatment, eggs were then incubated at a standard 37.5°C to determine subsequent effects on embryonic development rate, survival, water loss, hatching, and embryonic oxygen consumption. Both quantity of heating degree hours during pre-incubation and specific thermal regime (oscillating vs. non-oscillating) profoundly affected important aspects of embryo survival and indices of development and growth Pre-incubation quail eggs showed a remarkable tolerance to constant high temperatures (up to 45°C), surviving for 4.5±0.3 days of subsequent incubation, but high oscillating pre-incubation temperature increased embryo survival (mean survival 12.2±1.8 days) and led to more rapid development than high constant temperature (maximum 38.5°C), even though both groups experienced the same total heating degree-hours. Oxygen consumption was ~200–300 μl O_2_^.^egg^.^min^-1^ at hatching in all groups, and was not affected by pre-incubation conditions. Oscillating temperatures, which are the norm for pre-incubation quail eggs in their natural habitat, thus enhanced survival at higher temperatures. However, a 5°C increase in pre-incubation temperature, which equates to the predicted long-term increases of 5°C or more, nonetheless reduced hatching rate by approximately 50%. Thus, while pre-incubation bobwhite eggs may be resiliant to moderate oscillating temperature increases, global warming will likely severely impact wild bobwhite quail populations, especially in their strongholds in southern latitudes.

## Introduction

Temperature influences the rates of virtually all developmental processes in bird embryos, including metabolism, development, and growth [1–3]. Thus, it is not surprising that, within a zone of thermal tolerance, higher mean temperatures result in higher avian embryonic growth rates than lower mean temperatures [4–13], albeit with some potentially negative effects on hatchability. Many birds—especially domesticated species—maintain the incubation temperature of their eggs relatively constant and close to optimal temperature (typically 35–38°C) through behavioral and physiological manipulations [14]. As a result, most studies evaluating temperature effects on avian development use a constant incubation temperature equal to mean daily-temperatures of a particular species’ natural environment [14].

For economically important species such as the domestic chicken (*Gallus gallus*) or turkey (*Meleagris gallopavo*), for example, constant temperatures during incubation represent the natural incubation conditions and certainly the commercial industry standard. Consequently, relatively few studies have used cyclic thermal protocols to evaluate hatchability or post-natal growth—e.g., [15]. However, constant (non-cyclic) incubation temperatures do not fully represent the naturally encountered daily thermal fluctuations that eggs of many precocial, non-domesticated birds. Some species may lay multiple eggs in a clutch (e.g., ducks, gallinaceous birds, and ratites), depositing eggs in their nest at a rate of one egg per day. In such species, true incubation by one or more parents does not begin until the penultimate or ultimate egg is laid (see [2] for review, [16]). Thus, for many precocial birds there is an extended *pre-incubation* period—the focal period of this study—where eggs that are laid first are unattended for the entire pre-incubation period and thus lack the thermal protection and stabilization provided by an incubating parent.

Pre-incubation egg temperatures closely follow what can be large diurnal thermal fluctuations of the environment [17]. The magnitude of the diurnal thermal fluctuations varies locally, seasonally, and from year to year, being for example typically greater during drought years than non-drought years. Pre-incubation egg temperatures can paradoxically reach actual incubation temperatures, which could begin development of the fertilized egg. In fact, potentially lethal temperatures have been measured in pre-incubation eggs found in ground-nests of precocial birds. For example, internal egg temperatures of pre-incubated northern bobwhite eggs (*Colinus virginianus*) in their nest but not yet being tended, followed ambient thermal fluctuations in non-drought years and often exceeded 40°C during peak thermal intensity [12, 17, 18]. In fact, bobwhite nest temperatures in drought conditions regularly peaked at 45°C and were recorded as high as 60°C [19]. Bobwhite eggs in ground nests could be severely impacted by further increases in temperature variability and magnitude [7]. Indeed, the impacts of climate change and local habitat warming, especially on the thermal biology of species including birds and their development, only continue to grow—for an entry into the voluminous and expanding literature see [20–24].

The objective of this study was thus to determine if pre-incubation is actually an unrecognized critical period for subsequent avian morphological and physiological development that can be revealed by experimental thermal protocols (e.g., [23]). Specifically, we investigated if exposure to diurnally oscillating temperatures or a constant temperature of equal heating degree-hours (the product of temperature (°C) X time (hours)) during pre-incubation affects subsequent morphological and physiological development of northern bobwhite embryos during their actual incubation period, the duration of incubation, the hatchability of eggs, and the hatching synchrony of eggs. We hypothesized that bobwhite embryos exposed to oscillating temperature treatments would exhibit a differential rate of development, metabolism, and hatching compared to those exposed to a constant temperature of the same mean value, and that an increased heat load would negatively affect development, metabolism and hatching success.

## Materials and methods

Fertilized northern bobwhite eggs were collected from captive-reared breeding pairs of flight ready birds at Lake Cumberland Game Bird Farm (Mill Springs, KY, USA). Eggs were packaged and shipped to the University of North Texas (Denton, USA) on the day of collection. Eggs arrived on-site within 2 business days with a written record of the date and time of egg collection. Upon arrival, bobwhite eggs were randomly divided into 5 groups of 15 eggs, placed on plastic egg trays blunt end up, and given a unique identifying number with an indelible marker. Each egg was then weighed to the nearest 0.01 g with a digital scale (Ohaus Explorer Pro, Pinebrook, NJ, USA) and placed into the assigned pre-incubation treatment.

Lake Cumberland Game Bird Farm was approved for egg production by the United States Department of Agriculture (USDA) and certified by the USDA National Poultry Improvement Plan. This research was approved by the University of North Texas Institutional Animal Use Care Committee, protocol # 0808.

### Pre-incubation protocol

Each of the 5 egg groups (N = 15 eggs per group) were assigned to a thermal chamber (G.Q.F. 1583 Hova-Bator with circulated air) with a unique experimental thermal protocol of oscillating or constant temperatures for a 12-d pre-incubation period. The groups consisted of a low and high oscillating temperature regime (LT_Osc_, HT_Osc_), a low and high constant temperature regime (LT_Const_, HT_Const_), and a commercial regime (T_Comm_) that was standard in the commercial poultry production industry [25]. The specifics of these incubation regimes are given in Table 1.

**Table 1:** Diel thermal regimes for oscillating and constant treatments for eggs of northern bobwhites during a 12-d pre-incubation period.

Time of Day	Oscillating Thermal Regime		Constant Thermal Regime		
	Low Temp (LT_Osc_)	High Temp (HT_Osc_)	LowTemp (LT_Const_)	High Temp (HT_Const_)	Commercial Temp (T_Comm_)
0000–0759	25.00°C	30.00°C	28.85°C	33.85°C	20.00°C
0800–1059	30.00°C	35.00°C	28.85°C	33.85°C	20.00°C
1100–1359	35.00°C	40.00°C	28.85°C	33.85°C	20.00°C
1400–1659	40.00°C	45.00°C	28.85°C	33.85°C	20.00°C
1700–2359	25.00°C	30.00°C	28.85°C	33.85°C	20.00°C

Peak temperatures of the low and high groups were selected based on thermal studies which described temperatures peaking at ≥40°C in non-drought years and ≥45°C in simulated drought years [17, 19], in Texas. The experimental temperature regime for each group (low and high) ensured that the temperature of the constant treatment was equivalent to the mean of the oscillating treatment, and heating degree-hours were equivalent within the low and high groups. A heating degree-hour was defined as 1°C above physiological zero (25°C for *Colinus virginianus*) [26] for 1 hour, such that 26°C for 1 h = 1 heating degree-hour, and 27°C for 1 h = 2 heating degree-hours. Thus, the mean temperature for the LT_Osc_ group was equal to that of the LT_Const_ group (28.85°C) and mean temperature for the HT_Osc_ group was equal to that of the H_Const_ group (33.85°C). Further, each low group (LT_Osc_ and LT_Const_) received 92.4 heating degree-hours per day, and each high group (HT_Osc_ and H_Const_) received 212.4 heating degree-hours per day, for the entire pre-incubation. The commercial regime (T_Comm_; 20°C) received no heating degree-hours per day, since pre-incubation temperatures did not reach physiological zero. Oscillating chambers were kept at base temperatures of 25°C (LT_Osc_) and 30°C (HT_Osc_) from midnight (0000h) to 0800h and were increased by 5°C at 0800h, 1100h, and 1400h. At 1700h, chamber temperatures were returned to their base temperature. These diurnal fluctuations were carried out each day of the 12-d pre-incubation period. The constant temperature chambers were kept at the intended temperatures throughout the entire 12-day pre-incubation period (Table 1). All thermal chambers maintained a relative humidity (RH) of 60% to minimize compounding factors with temperature treatments. Eggs were not turned during this pre-incubation period.

### Assessment of development during pre-incubation

To establish a baseline of pre-incubation development for egg groups subjected to each pre-incubation thermal regime, and to investigate whether various pre-incubation thermal groups affected subsequent embryonic development, eggs from each group were randomly selected for analysis (23 eggs per thermal regime group were selected over the duration of the study). Following the pre-incubation protocol, on the morning of the 13^th^ day of incubation, selected eggs were weighed to the nearest 0.01 g to determine the amount of water loss during pre-incubation. Further, eggs were opened and if embryos were present, they were separated from the egg (yolk-free), weighed to the nearest 0.01 g, aged, and staged according to morphological indicators of development [27, 28].

### Incubation protocols

After the pre-incubation period, on the morning of the 13^th^ day, eggs from each group were removed from the thermal chamber, weighed to the nearest 0.01 g, and immediately placed into the incubator (G.Q.F. 1502 Sportsman incubator, G.Q.F. Manufacturing Co., Savannah, GA, USA). Incubator temperature was maintained at a constant 37.5±0.5°C with a RH of 60%. Eggs were turned automatically every 3 h for the first 19 days of the 23 day incubation [1]. On day 20 (D20) of incubation, eggs were weighed to the nearest 0.01 g and placed in the hatching chamber of the same incubator and egg turning was stopped [1]. Hatching was determined when eggs were star-pipped, defined as an externally pipped egg where the embryo created a small hole in the shell (approximately 3 mm^2^) to initiate hatching [24]. This definition of hatching was used to compensate for any artificial hatching difficulties. Upon star-pipping, the duration of incubation, degree of hatching synchrony, and percentage of eggs hatched was recorded. The stage at the time of death was recorded for eggs that did not hatch. A subset of incubating eggs was allowed to go to hatching, and the hatchlings were subsequently maintained for one day at 37.5±0.5°C with a RH of 60%, for oxygen consumption measurements (see below).

### Oxygen consumption

During incubation, on incubation days 10, 12, 14, 16, 18, 20, 22, and on 1-d post hatch, VO_2_ of the embryos *in ovo* was recorded via flow-through respirometry as an indicator of development and timing of physiological processes (e.g., internal pipping). On these selected days, 6 eggs from an individual group were removed and immediately placed into individual metabolic chambers located in a modified incubator (37.5°±0.5°C) as part of the flow-through respirometry system.

The respirometry system was operated by pumping compressed normoxic air (159.22 mmHg) into an airflow manifold (MF-8 Sable Systems International, Las Vegas, NV, USA) which split the airflow into 7 channels and set the rate air flow through the system at 100±1 ml·min^-1^ [28]. The seven Nalgene air tubes then went into the incubator in a sealed opening and connected to copper coils of the same diameter, to warm the air to incubation temperature. Air then flowed through Nalgene tubes connected the copper coils, then to the 7 metabolic chambers (modified 2.5 oz sterilized Gerber baby food jar) simultaneously. Chamber 1 was kept empty (the “blank”) and used as a reference for normoxic air, while chambers 2–7 contained eggs. From each chamber, air flowed to a multiplexor (Sable Systems International, Las Vegas, NV, USA) that regulated the sequence of airflow from the chambers to a column of Drierite and soda lime, which removed water vapor and carbon dioxide, respectively. A sub-sample pump then pulled a low-pressure air sample of each chamber, sequentially through the oxygen analyzer (FC-1B, Sable Systems International, Las Vegas, NV, USA). Each chamber was sampled at 5 sec consecutive intervals for 15 min. Oxygen measurements from the analyzer were sent to the computer via a Sable Systems Universal Interface II and processed by system software (Data acquisition system 2.0 and Datacan V, Sable Systems International, Las Vegas, NV, USA). VO_2_ was measured as the difference in oxygen concentration between the normoxic air flowing into the blank chamber (chamber 1) and the expired air flowing out of each treatment chamber (chambers 2–7).

### Statistical analyses

All data was tested with a Shapiro–Wilks normality test [29] and Hartley’s Fmax test [29] before specific statistical analyses were performed. An independent t-test [29] was used to compare oscillating to constant temperature egg groups. An ANOVA [29] was used to identify the relationship between groups for all experiments. A Kaplan-Meier survival analysis [30] was used to compare survival rates and time to mortality among groups. Significance between groups was determined with a Student–Newman–Keuls (SNK) multiple range *post hoc* test [29]. All statistical tests were conducted using SigmaStat 3.5 software (Systat Software Inc. San Jose, CA), or manually [29, 31]. Statistical decisions were made with a 0.05 level of probability. All data are reported as mean±S.E. unless otherwise indicated.

## Results

### Pre-incubation development

**Fig 1:**
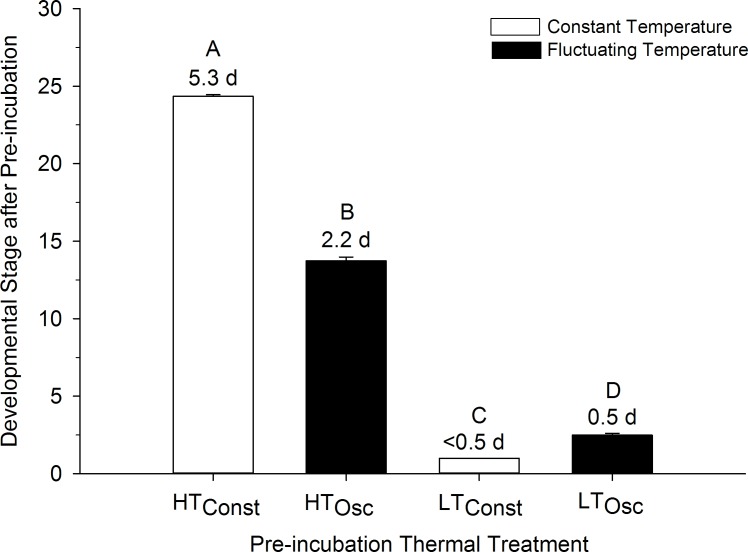
Mean developmental stages of northern bobwhite embryos after exposure to specific thermal groups during a 12-d pre-incubation period. Numbers above bars show days of incubation equivalent to the indicated developmental stages. Letters indicate statistical groupings. n = 23 eggs per group. Commercially incubated eggs did not have any pre-incubation development.

For the subsample of embryos extracted directly after the 12-d pre-incubation period, mean stages of development were different among pre-incubation treatments (ANOVA, P<0.001). Oscillating and constant groups significantly differed from each other (Fig 1) and developmental stage of all groups were statically unique (P<0.05). During pre-incubation, eggs of the high-constant temperature (H_Const_) group were the most developed, advancing to a mean developmental stage of 21.4±0.2, equivalent to 5.3 incubation days at 37.5°C [27, 28]. Eggs exposed to high-oscillating temperatures (HT_Osc_) developed to a mean stage of 13.7±0.2, equivalent to 2.2 incubation days at 37.5°C. Low-oscillating (LT_Osc_) embryos developed to a mean stage of only 2.5±0.1, equivalent to 0.5 incubation days at 37.5°C, while pre-incubation development was lowest in low-constant (LT_Const_) eggs, which progressed to a mean stage of just 1.1±0.1, or <0.5 incubation days at 37.5°C (Fig 1). Commercial pre-incubation eggs (T_Comm_) had no pre-incubation development.

### Survival curves hatching success

**Fig 2:**
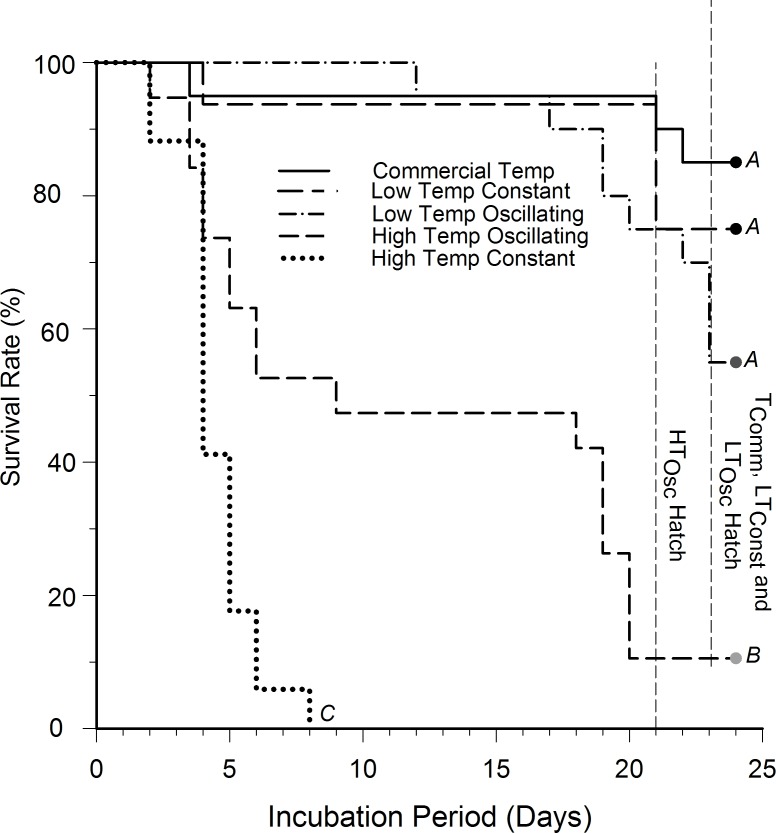
Kaplan-Meier survival curves showing survival rate and time to mortality of northern bobwhite embryos exposed to different thermal protocols during a 12-day pre-incubation period. Stair-step declines show time of mortality. n = 45 eggs per treatment group. Letters indicate statistical groupings with a P<0.05 level of significance.

Mortality occurred prior to hatching in all groups including the commercial group, as anticipated. The survival rate and stage of development at the time of death was significantly different between groups (Log rank survival analysis; P<0.001). A Holm-Sidak Pairwise MCP statistically grouped the treatments receiving the lowest amounts of heating degree-hours (LT_Osc_, LT_Const_, and T_Comm_) into one group. These groups had a statistically higher survival rate compared to the high heating degree-hour groups (HT_Osc_ and H_Const_; P<0.001, Fig 2). Mean survival time for LT_Const_ eggs was 22.2±1.4 days. The low oscillating group had a mean survival time of 22.1±0.7 days, and this was not statistically different from the LT_Const_ group (P = 0.25). Mean survival for HT_Osc_ eggs was 12.2±1.8 days which was significantly different from H_Const_ eggs (4.47±0.3 days), none of which hatched during any trials (Fig 2).

**Fig 3:**
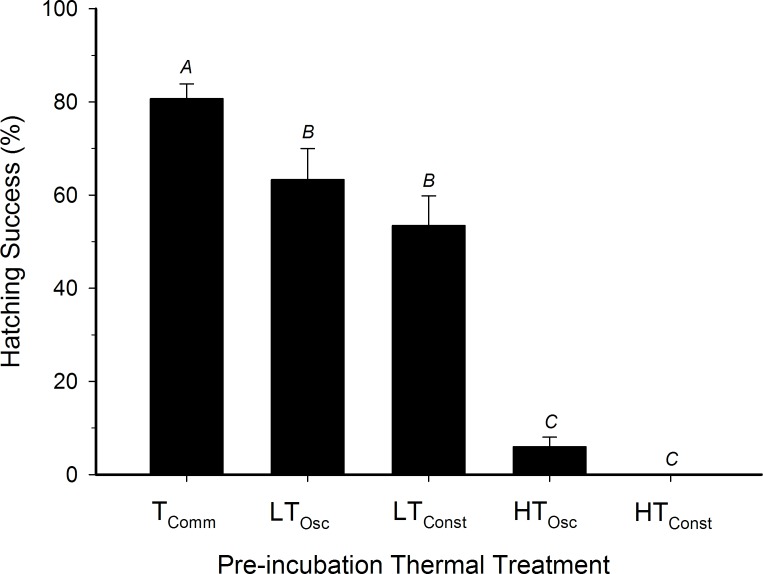
Hatching success of northern bobwhite embryos exposed to different thermal protocols during a 12 day pre-incubation period. n = 45 eggs per group. Letters indicate statistical groupings.

Commercial eggs hatched at a significantly higher rate (80.8±3.6%) than all treatment groups (Fig 3; ANOVA, P<0.001). Hatching success of the LT_Const_ treatment (63.3±6.7%) was not significantly different from the LT_Osc_ treatment (53.5±6.4%; P = 0.24). HT_Osc_ hatch rates (6.0±2.1%) did not statistically differ (P = 0.53) from H_Const_ hatch rates (0.0%). Hatch rates for LT_Osc_ was significantly higher than for the HT_Osc_ groups (P<0.001).

**Fig 4:**
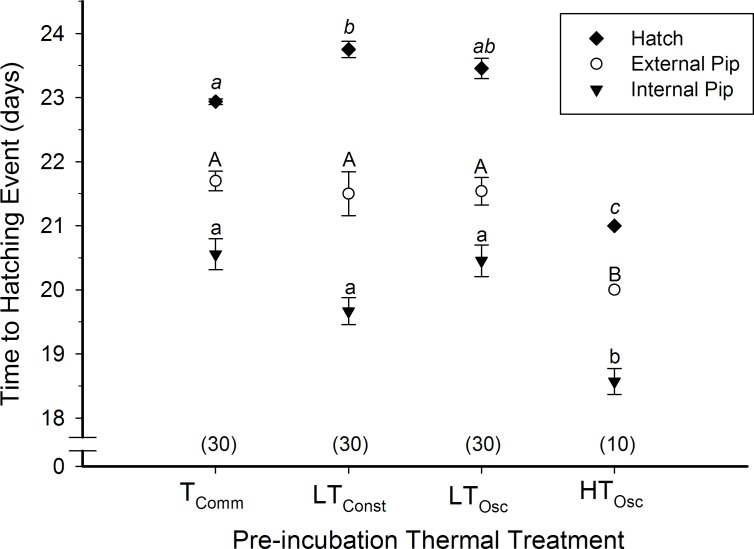
Time to internal and external pipping and hatching for northern bobwhite eggs exposed to T_Comm_, LT_Const_, LT_Osc_, and HT_Osc_ thermal treatments during a 12 day pre-incubation period. Sample size in parentheses. Events are statistically grouped (0.05 level of significance) with lower-case, italicized, and capital lettering.

Pre-incubation thermal treatment not only affected survival and hatching success, but also the timing of specific hatching events—internal pipping, external pipping, and hatching. All of these developmental landmarks were significantly different between commercial and treatment groups (Fig 4; ANOVA, P<0.001). HT_Osc_ eggs internally pipped earlier (D18.6±0.2) than LT_Const_ eggs (D19.7±0.2), LT_Osc_ eggs (D20.5±0.2), and T_Comm_ eggs (D20.6±0.2). Similarly, HT_Osc_ eggs externally pipped earlier (D20.0±0.0) than LT_Const_ eggs (D21.5±0.3), LT_Osc_ eggs (D21.5±0.2), and T_Comm_ eggs (D21.7±0.2). HT_Osc_ eggs also hatched earlier (D21.0±0.0) than T_Comm_ eggs (D23.0±0.0) and LT_Osc_ eggs (D23.5±0.2), while LT_Const_ eggs took the longest to hatch of treatment groups (D23.8±0.1).

Additionally, the duration from internal pip to hatching was significantly different between treatment groups (P<0.001) (Fig 4). The hatching duration was not significantly different between LT_Const_ (3.7±0.4 days) and LT_Osc_ (3.2±0.1 days) treatments (P = 0.16), but HT_Osc_ had a shorter duration of hatching events than LT_Osc_ (P<0.001). The duration between internal pip and hatch was the shortest for HT_Osc_ (2.4±0.2 days) and T_Comm_ eggs (2.4±0.2 days. All eggs in a given group hatched essentially synchronously–i.e., within 24 h of each other.

### Oxygen consumption

**Fig 5:**
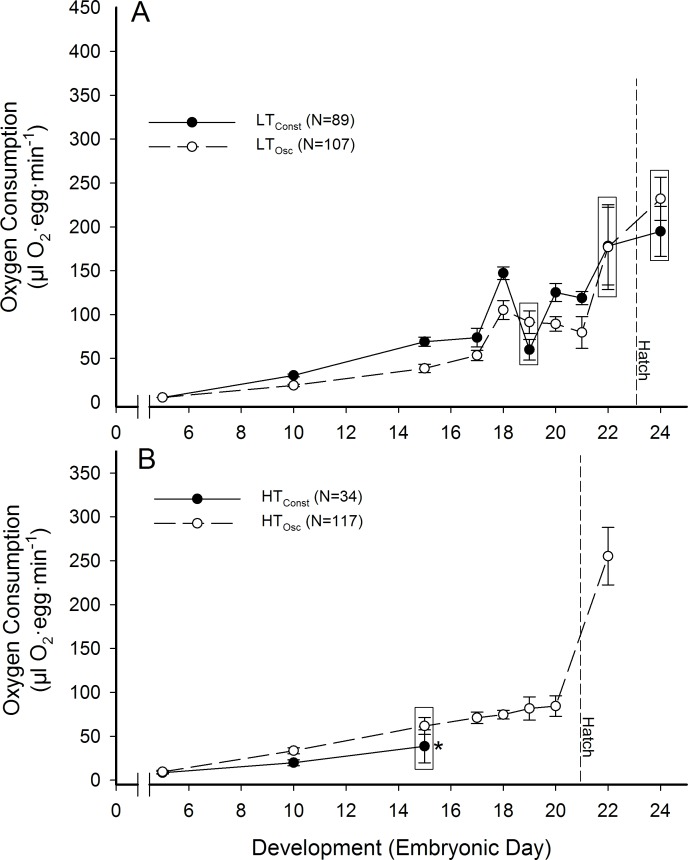
Oxygen consumption of northern bobwhite embryos as a function of development and pre-incubation thermal groups. Statistically similar means are grouped within boxes. * No H_Const_ eggs survived past day 15.

Oxygen consumption (VO_2_) increased significantly as a function of development (Fig 5). VO_2_ in LT_Const_ and LT_Osc_ groups was similar in overall pattern throughout incubation, but absolute values differed significantly (P<0.05) on D10, D15, D18, D20, and D21 of incubation (Fig 5A). HT_Osc_ embryos had a significantly higher VO_2_ than H_Const_ embryos on D10 but not on D15, however H_Const_ embryos did not survive past day 15 of incubation, so no further comparisons were possible (Fig 5B). Hatchling VO_2_ was higher than at any point in embryonic development in all groups, but there were no significant differences between treatments in hatchlings.

**Fig 6:**
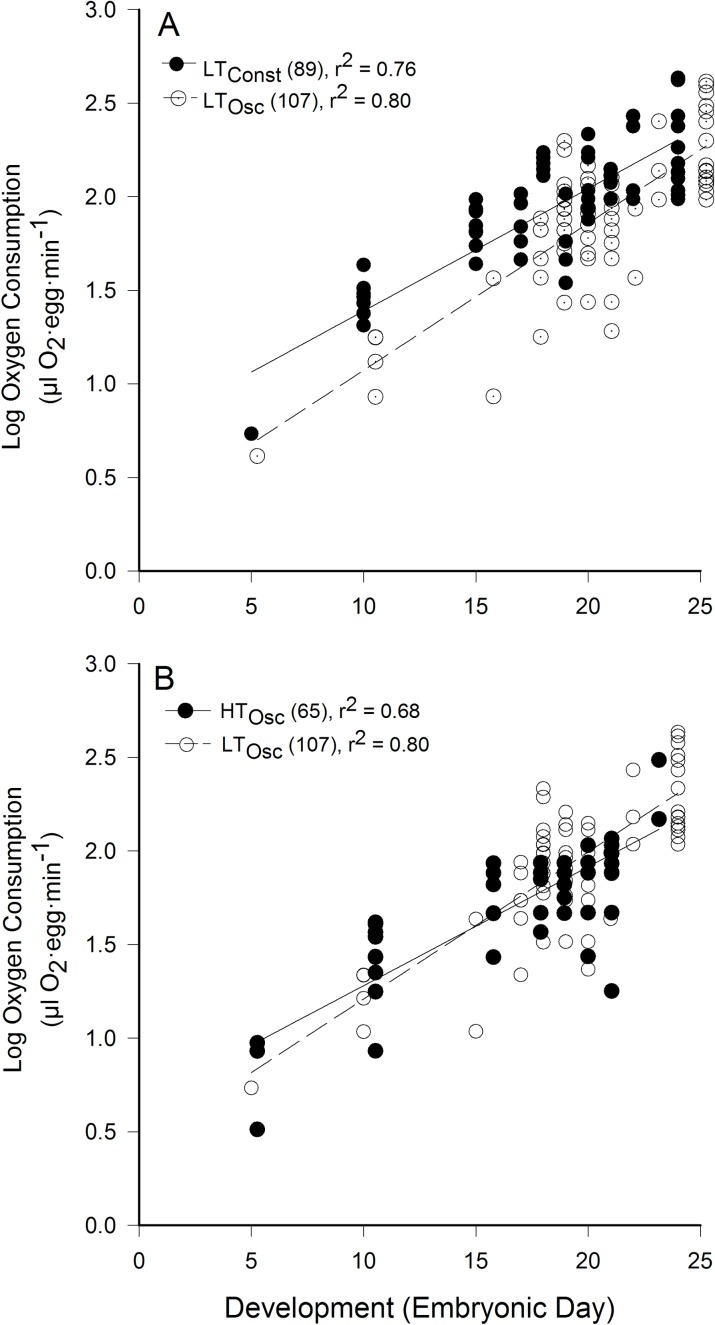
Regressions relating oxygen consumption to development. A) No significant differences existed between the slope of the lines for LT_Const_ and LT_Osc_ (ANOVA; P>0.50). B) The slope of the line for relating LT_Osc_ was significantly higher than for HT_Osc_ (0.02>P>0.001). Coefficient of determination (r^2^) and n values are shown.

To further aid in VO_2_ comparison, semi-log plots were created (Fig 6). Slopes relating VO_2_ to development were not significantly different between LT_Const_ and LT_Osc_ pre-incubation treatments (Fig 6A; t test for slopes, P>0.50). Slopes relating VO_2_ to development were significantly different between LT_Osc_ and HT_Osc_ pre-incubation treatments (Fig 6B; t-test for slopes, 0.02>P>0.001). As noted above, H_Const_ eggs did not survive past day 15 of incubation, so no oxygen consumption comparisons were made with this treatment group.

### Egg mass and water loss

**Fig 7:**
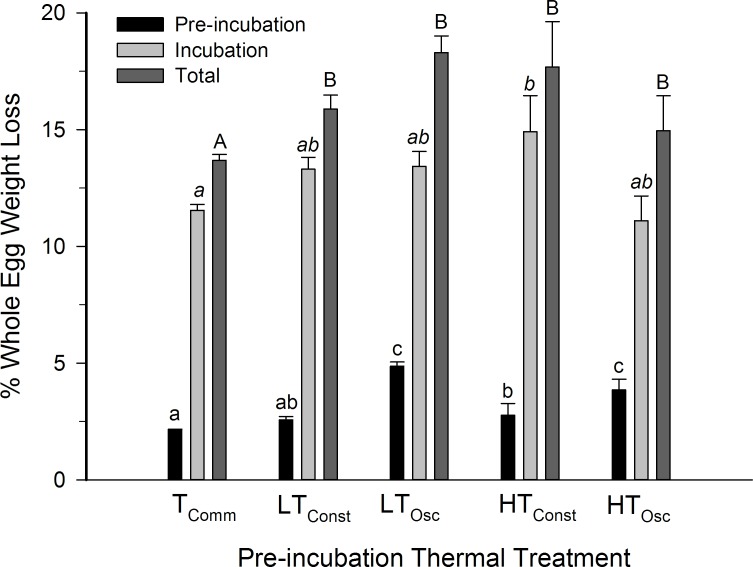
Mean percentage of fresh egg weight loss for northern bobwhite eggs measured after exposure to oscillating and constant thermal treatment for a 12 day pre-incubation period. n = 45 eggs per group. Letters (lower-case, italicized, and capital) indicate statistical groupings within each treatment.

Mean egg weight loss among thermal groups during the pre-incubation period was higher in LT_Osc_ and HT_Osc_ egg groups than in all other pre-incubation groups (P<0.001), with a percentage loss of 4.9±0.2% and 3.9±0.5% respectively. There was no difference between HT_Const_ eggs (2.8±0.5%) and LT_Const_ eggs (2.6±0.1%) and between LT_Const_ eggs and T_Comm_ eggs (2.14±0.04%; P>0.05) (Fig 7).

Unlike pre-incubation, no significant difference (P = 0.88) in the percentage of fresh egg weight loss during incubation was detected between HT_Osc_ and H_Const_ (P = 0.05) or LT_Osc_ and LT_Const_ (P = 0.88) groups. However, there was a significant (P<0.05).difference in egg weight loss between T_Comm_ eggs (11.5±0.3%) and H_Const_ eggs (14.9±1.5%) during incubation (Fig 7).

Combining the pre-incubation and incubation periods, all treatment groups lost a greater percentage of fresh egg mass than T_Comm_ eggs (ANOVA, P<0.05). The mean percentage of fresh egg mass lost by T_Comm_ eggs eggs was 11.5±0.3%, lower than H_Const_ (17.7±1.9%), HT_Osc_ (14.9±1.5%), LT_Const_ (15.9±0.6%), and LT_Osc_ eggs (18.3±0.7%). No difference was detected in percentage of fresh egg mass lost between HT_Osc_ and H_Const_ (P = 0.28), or LT_Osc_ and LT_Const_ (P = 0.12) (Fig 7).

## Discussion

### Pre-incubation modification of subsequent embryonic development

This study shows that quail embryos actually begin to develop during pre-incubation when eggs are exposed to temperatures above “physiological zero”. As expected, more development was obtained with more heating degree-hours and less development occurred with fewer heating degree-hours, even when slightly above physiological zero. Eggs exposed to high heat loads (HT_Osc_ and H_Const_) and low heat loads (LT_Osc_ and LT_Const_) had equal quantities of heating degree-hours within groups, but they all exhibited differential pre-incubation development and growth rates. Groups exposed to a constant temperature had the largest variation between high and low groups, with a difference of ~5 days of development between H_Const_ and LT_Const_, even though each group received the same amount of heating degree-hours (Fig 1). The difference in groups with oscillating temperature was much less, with about 1.7 days of development difference between HT_Osc_ and LT_Osc_. Thus, at least for quail development, the quantity of heat (e.g., number of heating degree-hours) is not the only driver of development and that the nature of the heat (oscillating or constant) is also a major factor.

Recently, intragenerational epigenetic modification of embryonic development has been induced by the length of pre-incubation storage in chicken embryos [32]. These changes were not the result of high temperatures, since the eggs were stored well below physiological zero. Whether an oscillating temperature at cool temperatures with no likelihood of beginning embryonic development would actually alter embryonic development remains to be investigated.

### Survival and hatching assessment

The thermal protocol used during the pre-incubation period in *Colinus virginianus* had a profound effect on subsequent embryonic survival. While high pre-incubation temperatures severely affected subsequent embryonic survival, it is interesting to note that an equivalently high oscillating temperature, while leading to only poor survival, nonetheless was more successful in producing viable embryos than a constant high temperature.

Observations of the effect of pre-incubation temperature and other conditions on subsequent embryonic survival have mostly derived from interest in poultry production or basic experimentation [32–38]. Such studies on the pre-incubation period have almost invariably considered steady-state storage conditions, rather than oscillations in environmental parameters, such as the oscillations in temperature employed in the current study. Few studies on oscillating temperatures exist for any aspect of avian egg storage—the few such studies have been directed towards inducing temperature oscillations once formal incubation has begun [15].

Embryonic survival and hatching success was not statistically different between constant and oscillating groups. However, H_Const_ embryos did not hatch, dying at ~4 to 15 days of incubation (Fig 2). The death of H_Const_ embryos is noteworthy, since eggs were kept for 12 days at 33.85°C, well within the natural incubating range of 30–35°C measured in nature for this species [17]. While the objective of this study was not to determine the cause of death in eggs at constant high temperature, we speculate that constant temperatures near incubation temperature require eggs to be turned daily for survival. Indeed, studies have shown that, while turning of quail eggs is not required when eggs are stored at 12.8°C, turning is required during incubation at temperatures between 35–38°C [1, 39, 40]. High mortality was not experienced in LT_Const_ groups (28.85°C) presumably because the mass of the embryo was that at <0.5 day of age (i.e. a much smaller embryo), and thus was not affected by the absence of turning.

Bobwhite quail eggs exposed to high oscillating temperatures internally pipped, externally pipped, and hatched two days earlier than all other groups, ultimately showing a reduced incubation period (Fig 4). This should not be surprising since the high oscillating temperature protocol (HT_Osc_) during pre-incubation enabled approximately 2 additional days of development than in LT_Osc_ and LT_Const_ groups. Both LT_Osc_ and LT_Const_ eggs, which developed approximately 0.5 days during the pre-incubation period, exhibited an extended incubation time of 0.5 d (Fig 4). HT_Osc_ eggs not only started development earlier, but also showed overall reduced incubation duration of HT_Osc_ eggs. This may be an adaptation to the thermally stressful environments inhabited by northern bobwhite quail, where both high temperature and temperature fluctuation are common and would affect the eggs during their pre-incubation period.

Collectively, our results suggest that oscillating temperatures act differently on developing organisms than constant temperatures of equal heating degree-hours. The impact of these differences is increased as heat loads are increased, i.e., higher heating degree-hours result in more variation in development in embryos exposed to constant and oscillating temperatures. Further, within oscillating groups, higher oscillating heat loads caused an increase in the rate of development during pre-incubation, a decrease in the rate of development during incubation, and had more of a negative effect on hatching.

### Hatching synchrony

While the current study did not focus on hatching synchronization, it is interesting to note that, irrespective of thermal regime, all quail eggs hatched within 24h of each other–i.e. “synchronously” [41]. Northern bobwhites do not begin incubation until the last egg is laid [42]. Consequently, all eggs receive equal amounts of incubation, resulting in synchronous hatching. This hatching adaptation seems beneficial by allowing the incubating parent to quickly transition behavior from incubating sessile eggs to brooding motile hatchlings [41, 43]. Importantly, limited studies have investigated hatching synchrony in warmer climates where nest temperatures fluctuate during pre-incubation and can often exceed physiological zero (the temperature above which embryonic development begins). In warmer climates, those eggs laid early could actually begin to develop prior to incubation, resulting in asynchronous development at the onset of incubation and asynchronous hatching at the other end of embryonic development. In this case, eggs might hatch asynchronously unless a synchronizing mechanism takes place [6, 44–46]. Hatching synchrony has been observed in the laboratory among quail eggs that differed in developmental stages by 2 days [46], but not by up to 5 days, which is the probable result of northern bobwhites in warm climates given our experimental findings on stage of development in high temperature groups at the onset of incubation. Interestingly, eggs must remain in physical contact with each other for synchronous hatching to occur, especially in the last 20% of incubation when pipping occurs [46, 47]. Regardless of relatedness or order of laying, viable bobwhite eggs of the same age hatch synchronously if touching [48]. When isolated, their time of hatch is spread over a longer period [45]. Bobwhite eggs accelerated hatching when placed in contact with more-advanced eggs (24 h advanced in development) and delayed hatching when placed in contact with more-retarded eggs (24 h retarded in development) [45], suggesting that both an acceleration and delay of hatching time occur between earlier laid and later laid eggs in the wild.

### Oxygen consumption and development

Oxygen consumption during avian embryonic development has been very well documented, and the reader is directed to recent discussions of that literature [16, 49–51]. Many factors influence avian embryonic VO_2_, most notably body mass, oxygen availability, and environmental temperature. Yet, in precocial birds, there is a representative pattern of VO_2_ change during development in which VO_2_ typically increases until approximately 80% of incubation time. Thereafter, rate of VO_2_ change plateaus until the embryo internally pips into the air cell. VO_2_ increases once more, followed by final increase at the time the chick pips the shell and hatches [47].

The oxygen consumption pattern of change in the current study (Fig 5) was generally that described for precocial birds [47], and the pattern is consistent with reports of developing northern bobwhite quail [52] as well as the Coturnix quail, *Coturnix coturnix* [41], a precocial bird with similar sized eggs. Importantly, in our study the thermal protocol during pre-incubation significantly affected VO_2_, which was different between LT_Const_ and LT_Osc_ thermal groups. The slopes of the normalized VO_2_ data were not different between LT_Const_ and LT_Osc_ treatments (Fig 6A), indicating no overall differences in rate of development to match the elevation of development induced by higher temperature.

There were significant differences in VO_2_ between HT_Osc_ and LT_Osc_ groups logically due to HT_Osc_ embryos having developed more during pre-incubation (Fig 1), thus requiring more oxygen on any given day during incubation. However, the lower slope of VO_2_ for HT_Osc_ eggs during incubation showed that HT_Osc_ eggs developed at a slower rate during incubation than LT_Osc_ eggs after a higher rate of development during pre-incubation (Fig 6B). Additionally, HT_Osc_ eggs did not exhibit the same oscillating VO_2_ patterns in the last 20% of incubation as LT_Osc_ and LT_Const_ eggs. Rather, VO_2_ of HT_Osc_ eggs stayed relatively constant during the pipping process (Fig 5B). Why and how VO_2_ during incubation is affected by pre-incubation thermal protocols awaits further investigation.

### Implications for natural bobwhite quail populations

The intention of comparing LT_Osc_ (simulated non-drought temperatures) and HT_Osc_ (simulated drought temperatures) groups was to better understand the differences between drought and non-drought conditions as well as the potential impacts of global warming on avian development generally, and quail populations specifically. In this context, one of the most interesting findings is the significant difference in hatch rates between high-oscillating (HT_Osc_, 6.0±2.1%) and low-oscillating (LT_Osc_, 53.5±6.4%) groups (Fig 2). The HT_Osc_ thermal group was pre-incubated at a temperature 5.0°C higher than the LT_Osc_ group, a temperature increase coincident with the predicted increase in global average temperature of 5±1°C in the United States by the period 2071–2100 relative to the period 1961–1990 [53]. Our results show that a 5°C increase in pre-incubation temperature of just 12 days reduces hatching rate of northern bobwhites by approximately one half. Reductions in the percentage of juvenile bobwhite quail at different lattitudes [54] or during drought [55, 56] could, according to our findings, be caused by increased heat loads during the 12 day pre-incubation period. Eggs in ground nests could be severely impacted by further increases in temperature variability and magnitude as global warming continues. Thus, our study indicates that the dynamic nature and magnitude of diel temperature should be considered when evaluating the biological response to predicted temperatures of global warming.

## References

[1] Romanoff A. The avian embryo New York, New York, USA: Macmillan; 1960.

[2] Tazawa H, Whittow GC. Incubation physiology In: Whittow GC, editor. Avian Physiology. 5th ed. San Diego, California, USA: Academic Press; 2000 p. 617–32.

[3] Starck JM, Ricklefs RE, editors. Avian growth and development: evolution within the altricial-precocial spectrum New York, NY USA: Oxford University Press; 1998.

[4] French NA. Modeling incubation temperature: the effects of incubator design, embryonic development, and egg size. Poultry science. 1997;76:124–33. 9037699 10.1093/ps/76.1.124

[5] Pendlebury CJ, MacLeod MG, Bryant DM. Variation in temperature increases the cost of living in birds. Journal of Experimental Biology. 2004 5 15, 2004;207(12):2065–70. 15143140 10.1242/jeb.00999

[6] Stoleson SH, Beissinger SR. Egg viability as a constraint on hatching synchrony at high ambient temperatures. J Anim Ecol. 1999;68:951–62.

[7] Reyna KS, Burggren WW. Upper lethal temperatures of Northern Bobwhite embryos and the thermal properties of their eggs. Poultry science. 2012 1;91(1):41–6. doi: 10.3382/ps.2011-01676 . 22184426 10.3382/ps.2011-01676

[8] Narinc D, Erdogan S, Tahtabicen E, Aksoy T. Effects of thermal manipulations during embryogenesis of broiler chickens on developmental stability, hatchability and chick quality. Animal: an international journal of animal bioscience. 2016 8;10(8):1328–35. doi: 10.1017/S1751731116000276 . Epub 2016/03/05. Eng. 26932726 10.1017/S1751731116000276

[9] Maatjens CM, van Roovert-Reijrink IA, Engel B, van der Pol CW, Kemp B, van den Brand H. Temperature during the last week of incubation. I. Effects on hatching pattern and broiler chicken embryonic organ development. Poultry science. 2016 4;95(4):956–65. doi: 10.3382/ps/pev447 . Epub 2016/01/21. Eng. 26787926 10.3382/ps/pev447

[10] Kosin IL. Studies on pre-inubation warming of chicken and turkey eggs. Poultry science. 1956;35:1384–92.

[11] Alsop FM. The effect of abnormal temperatures upon the developing nervous system in the chick embryos. The Anatomical record. 1919;15(6):306–31.

[12] Decuypere E, Michels H. Incubation temperature as a management tool: a review. World's Poultry Science Journal. 1992;48(1):28–38.

[13] Oppenheim RW, Levin HL. Short-term changes in incubation temperature: behavioral and physiological effects in the chick embryo from 6 to 20 days. Developmental psychobiology. 1974;8(2):103–15. 10.1002/dev.420080203 1225687

[14] Webb DR. Thermal tolerance of avian embryos: A review. The Condor. 1987;89:874–98.

[15] Loyau T, Bedrani L, Berri C, Metayer-Coustard S, Praud C, Coustham V, et al Cyclic variations in incubation conditions induce adaptive responses to later heat exposure in chickens: a review. Animal: an international journal of animal bioscience. 2015 1;9(1):76–85. doi: 10.1017/S1751731114001931 . Epub 2014/08/15. Eng. 25118598 10.1017/S1751731114001931

[16] Mueller CA, Burggren WW, Tazawa H. The Physiology of the Avian Embryo In: Whittow GC, editor. Sturkie's Avian Physiology. 6th ed. New York: Elsevier; 2015 p. 739–66.

[17] Guthery FS, Rybak AR, Fuhlendorf SD, Hiller TL, Smith SG, Puckett WH Jr., et al Aspects of the thermal ecology of bobwhites in north Texas. Wildlife Monographs. 2004;159:1–36.

[18] Gill FB. Ornithology. 2nd ed. New York: W. H. Freeman and Company; 1999.

[19] Guthery FS, Forrester ND, Nolte KR, Cohen WE, Kuvlesky J, W. P., editors. Potential effects of global warming on quail populations. Quail IV: Fourth National Quail Symposium; 2000; Tall Timbers Research Station, Tallahassee, Florida.

[20] Lawson CR, Vindenes Y, Bailey L, van de Pol M. Environmental variation and population responses to global change. Ecol Lett. 2015 7;18(7):724–36. doi: 10.1111/ele.12437 . Epub 2015/04/23. Eng. 25900148 10.1111/ele.12437

[21] Diamond SE. Evolutionary potential of upper thermal tolerance: biogeographic patterns and expectations under climate change. Annals of the New York Academy of Sciences. 2016 10 5 doi: 10.1111/nyas.13223 . Epub 2016/10/06. Eng. 27706832 10.1111/nyas.13223

[22] Dillon ME, Woods HA, Wang G, Fey SB, Vasseur DA, Telemeco RS, et al Life in the Frequency Domain: the Biological Impacts of Changes in Climate Variability at Multiple Time Scales. Integr Comp Biol. 2016 7;56(1):14–30. doi: 10.1093/icb/icw024 . Epub 2016/06/03. Eng. 27252201 10.1093/icb/icw024

[23] El-Tarabany MS. Effect of thermal stress on fertility and egg quality of Japanese quail. J Therm Biol. 2016 10;61:38–43. doi: 10.1016/j.jtherbio.2016.08.004 . Epub 2016/10/08. eng. 27712658 10.1016/j.jtherbio.2016.08.004

[24] Alkan S, Karslı T, Karabağ K, Galic A, Balcıoğlu MS. The effects of thermal manipulation during early and late embryogenesis on hatchability, hatching weight and body weight in Japanese quails (*Coturnix coturnix japonica*). Arc Tierz 2013;18:789–96.

[25] Dozier W, Bramwell K, Hatkin J, Dunkley C. Bobwhite quail production and management guide. Athens, GA2010.

[26] Miller ER, Wilson HR. The temperature required to initiate blastoderm development of bobwhite quail eggs. Poultry science. 1975;54:901–5. 1153384 10.3382/ps.0540901

[27] Hendrix AG, Hanzlik R. Developmental stages of the bob-white quail embryo (*Colinus virginianus*). Biological Bulletin. 1965 12 1, 1965;129(3):523–31. doi: 10.2307/1539730 5849122 10.2307/1539730

[28] Hamburger V, Hamilton HL. A series of normal stages in the development of the chick embryo. J Morphol. 1951;88:49–92. 24539719

[29] Zar JH. Biostatistical analysis 4th ed. Upper Saddle River, New Jersey, USA: Prentice Hall; 1999.

[30] Kaplan EL, Meier P. Nonparametric estimation from incomplete observations. Journal of American Statistical Association. 1958;53:457–81.

[31] Beitinger TL. Biostatistics helpbook. 2006.

[32] Branum SR, Tazawa H, Burggren WW. Phenotypic developmental plasticity induced by preincubation egg storage in chicken embryos (Gallus gallus domesticus). Physiological reports. 2016 2;4(4). doi: 10.14814/phy2.12712 . Epub 2016/02/26. eng. 26908714 10.14814/phy2.12712 PMC4816897

[33] Arora KL, Kosin IL. Developmental responses of early turkey and chicken embryos to preincubation holding of eggs: inter- and intra-species differences. Poultry science. 1966 9;45(5):958–70. . Epub 1966/09/01. Eng. 6008255 10.3382/ps.0450958

[34] Fasenko GM. Egg storage and the embryo. Poultry science. 2007 5;86(5):1020–4. . Epub 2007/04/17. eng. 17435042 10.1093/ps/86.5.1020

[35] Hamidu JA, Uddin Z, Li M, Fasenko GM, Guan LL, Barreda DR. Broiler egg storage induces cell death and influences embryo quality. Poultry science. 2011 8;90(8):1749–57. doi: 10.3382/ps.2011-01361 . Epub 2011/07/15. eng. 21753212 10.3382/ps.2011-01361

[36] Mather CM, Laughlin KF. Storage of hatching eggs: the effect on total incubation period. British Poultry Science. 1976;17:471–9.

[37] Mayes FJ, Takeballi MA. Storage of the eggs of the fowl (Gallus domesticus) before incubation: a review. World's Poultry Science Journal. 1984;40:131–40.

[38] Reijrink IA, Berghmans D, Meijerhof R, Kemp B, van den Brand H. Influence of egg storage time and preincubation warming profile on embryonic development, hatchability, and chick quality. Poultry science. 2010 6;89(6):1225–38. doi: 10.3382/ps.2009-00182 . Epub 2010/05/13. eng. 20460670 10.3382/ps.2009-00182

[39] Proudfoot FG. The handling and storage of hatching eggs In: Carter TC, Freeman BM, editors. The fertility and hatchability of the hen's egg. Edinburgh, UK: Oliver and Boyd; 1969 p. 127–41.

[40] Wilson HR. Physiological requirements of the developing embryo: termperature and turning In: Tullett SG, editor. Avian Incubation. Cambridge, UK: Butterworth-Heinemann Ltd; 1991 p. 145–56.

[41] Vleck CM, Vleck D, Hoyt DF. Patterns of metabolism and growth in avian embryos. American Zoologist. 1980 1 1, 1980;20(2):405–16.

[42] Stoddard HL. The bobwhite quail; its habits, preservation and increase New York, New York, USA: Scribner; 1931.

[43] Warkentin KM. Oxygen, gills, and embryo behavior: mechanisms of adaptive plasticity in hatching. Comparative Biochemistry and Physiology Part A. 2007. 10.1016/j.cbpa.2007.02.009 17363310

[44] Vince MA. Synchronization of hatching in American bobwhite quail (*Colinus virginianus*). Nature. 1964;203:1192–3. 14213686 10.1038/2031192a0

[45] Vince MA. Retardation as a factor in the synchronization of hatching. Animal Behavior. 1968;16:332–5. 10.1016/0003-3472(68)90017-1 5674238

[46] Vince MA, Ockelford E, Reader M. They synchronisation of hatching in quail embryos: aspects of development affected by a retarding stimulus. Journal of Experimental Zoology. 1984;229:273–82. doi: 10.1002/jez.1402290213 6736887 10.1002/jez.1402290213

[47] Visschedijk HHJ. The air space and embryonic respiration. I. The pattern of gaseous exchange in the fertile egg during the closing stages of incubation. British Poultry Science. 1968;9:173–84. doi: 10.1080/00071666808415707 5650818 10.1080/00071666808415707

[48] Pani PK, Coleman TH, Georgis HD, Kulenkamp AW. Between family synchronisation of hatching time in bobwhite quail. Poultry science. 1969;48:665–7. 5355512 10.3382/ps.0480665

[49] Haron A, Dahan Y, Shinder D, Druyan S. Physiological effects of hypoxic conditions during the plateau period on the chicken embryo. Comparative biochemistry and physiology Part A, Molecular & integrative physiology. 2016 8 21;203:32–9. doi: 10.1016/j.cbpa.2016.08.015 . Epub 2016/10/28. Eng. 27557989 10.1016/j.cbpa.2016.08.015

[50] Alvine T, Burggren WW. Renal, metabolic and hematological effects of trans-retinoic acid during critical developmental windows in the embryonic chicken. Journal of Comparative Physiology B. 2014 1;184(1):107–23. doi: 10.1007/s00360-013-0777-9 . 24005719 10.1007/s00360-013-0777-9

[51] Mortola JP, Marinescu DC, Pierre A, Artman L. Metabolic and heart rate responses to hypoxia in early chicken embryos in the transition from diffusive to convective gas transport. Respiratory physiology & neurobiology. 2012 4 30;181(2):109–17. doi: 10.1016/j.resp.2012.02.002 . Epub 2012/03/01. eng. 22366866 10.1016/j.resp.2012.02.002

[52] Williams JB, Swift K. Oxygen consumption and growth of northern bobwhite embryos under normoxic and hyperoxic conditions. The Condor. 1988;90(1):187–92.

[53] IPCC. Climate change 2014: Impacts, adaptation and vulnerability Cambridge, UK: Cambridge University Press, 2014.

[54] Guthery FS. On bobwhites College Station, Texas, USA: Texas A&M University Press; 2000.

[55] Bridges AS, Peterson MJ, Silvy NJ, Smeins FW, Wu B. Differential influence of weather on regional quail abundance in Texas. Journal of Wildlife Management. 2001;65:10–8.

[56] Reyna K, Rollins D, Ransom D. The Texas Quail Index: Evaluating Predictors Of Northern Bobwhite Productivity And Abundance Using Citizen Science. Proceedings of the National Quail Symposia. Knoxville, TN2012. p. 138–46.

